# Titanium dioxide nanoparticles enhance thrombosis through triggering the phosphatidylserine exposure and procoagulant activation of red blood cells

**DOI:** 10.1186/s12989-021-00422-1

**Published:** 2021-08-04

**Authors:** Yiying Bian, Han-Young Chung, Ok-Nam Bae, Kyung-Min Lim, Jin-Ho Chung, Jingbo Pi

**Affiliations:** 1 School of Public Health, China Medical University, Shenyang, 110122 People’s Republic of China; 2 Department of Agricultural Biotechnology, and Center for Food Safety and Toxicology, Seoul National University, Seoul, 151-742 South Korea; 3 College of Pharmacy, Hanyang University, Ansan, Gyeonggido 426-791 South Korea; 4 College of Pharmacy, Ewha Womans University, Seoul, 120-750 South Korea; 5 College of Pharmacy, Seoul National University, Seoul, 151-742 South Korea

**Keywords:** Titanium dioxide nanoparticles (TiO_2_ NPs), Phosphatidylserine (PS) exposure, Procoagulant activity, Thrombosis, Red blood cells (RBCs)

## Abstract

**Background:**

Expanding biomedical application of anatase titanium dioxide (TiO_2_) nanoparticles (NPs) is raising the public concern on its potential health hazards. Here, we demonstrated that TiO_2_ NPs can increase phosphatidylserine (PS) exposure and procoagulant activity of red blood cells (RBCs), which may contribute to thrombosis.

**Results:**

We conducted in vitro studies using RBCs freshly isolated from healthy male volunteers. TiO_2_ NPs exposure (≦ 25 μg/mL) induced PS exposure and microvesicles (MV) generation accompanied by morphological changes of RBCs. While ROS generation was not observed following the exposure to TiO_2_ NPs, intracellular calcium increased and caspase-3 was activated, which up-regulated scramblase activity, leading to PS exposure. RBCs exposed to TiO_2_ NPs could increase procoagulant activity as measured by accelerated thrombin generation, and enhancement of RBC-endothelial cells adhesion and RBC-RBC aggregation. Confirming the procoagulant activation of RBC in vitro, exposure to TiO_2_ NPs (2 mg/kg intravenously injection) in rats increased thrombus formation in the venous thrombosis model.

**Conclusion:**

Collectively, these results suggest that anatase TiO_2_ NPs may harbor prothrombotic risks by promoting the procoagulant activity of RBCs, which needs attention for its biomedical application.

**Supplementary Information:**

The online version contains supplementary material available at 10.1186/s12989-021-00422-1.

## Background

In addition to the wide use in sunscreens, titanium dioxide nanoparticles (TiO_2_ NPs) are receiving an increasing attention for biomedical applications like cell imaging, biological analysis, drug delivery and photodynamic therapy owing to their excellent and unique photocatalytic properties, and good biocompatibility [1–5]. Due to their wide and heavy uses in human life, concern of health hazard of TiO_2_ NPs is escalating and many researches have illuminated that TiO_2_ NPs can induce various pathological alterations in liver, spleen, kidneys and brain [6]. Meanwhile, the toxicity of TiO_2_ NPs on blood cells, the primary target cells of intravenously given substances, remains relatively unillustrated.

Indeed, previous studies demonstrated that some nanoparticles can inflict cytotoxicity and genotoxicity on lymphocyte cells [7, 8] and activate platelets, resulting in thrombosis [9, 10], reflecting that blood cells can be an important target of toxicity for nanoparticles. RBCs are also reported as a target of nanoparticles but the effects are mainly limited to hemolysis, morphological alterations or RBC aggregation [11–13]. Recently, an active role of RBCs in the development of thrombotic diseases has been demonstrated. Participating in thrombosis, RBCs accelerate the cascade of coagulation and the formation of blood clotting through externalization of phosphatidylserine (PS) on the outer membrane providing a procoagulant sites and faciliating thrombin generation [14]. This process is called as the procoagulant activity of RBCs, which is triggered by the perturbation of membrane phospholipid translocases; scramblase and flippase. Perturbation of membrane phospholipid translocases are caused by upstream events of intracelluar calcium increase, caspase activation, ROS production as well as ATP- and thiol-depletion [15–21].

Our previous study firstly demonstrated that the procoagulant activity of RBCs can be induced by silver nanoparticles, enhancing thrombosis [22], reflecting the role of RBCs in prothrombotic effects of nanoparticles. Previous studies showed that TiO_2_ NPs can induce hemolysis, morphological observation and a possible interaction with RBCs via penetration [7, 23, 24], implying their potental effects on RBCs with respect to procoagulant activity and thrombosis [23]. However, previous studies have not further extended into the investigation of the possible effects of TiO_2_ NPs on the development of procoagulant activity of RBCs and thrombosis.

Here, we examined whether TiO_2_ NPs can affect PS exposure and procoagulant activity of RBCs. In addition, we clarified the underlying mechanism and investigated the biological significances by evaluating the procoagulant activity of RBCs, RBC adhesion to endothelial cells and RBC aggregates. Importanly, the signficance of these findings with respect to human thrombosis were further substantiated by in vivo thrombosis using a rat venous thrombosis model.

## Results

### Characterization of TiO_2_ NPs and TEM analysis of TiO_2_ NPs-exposed RBCs

**Fig. 1:**
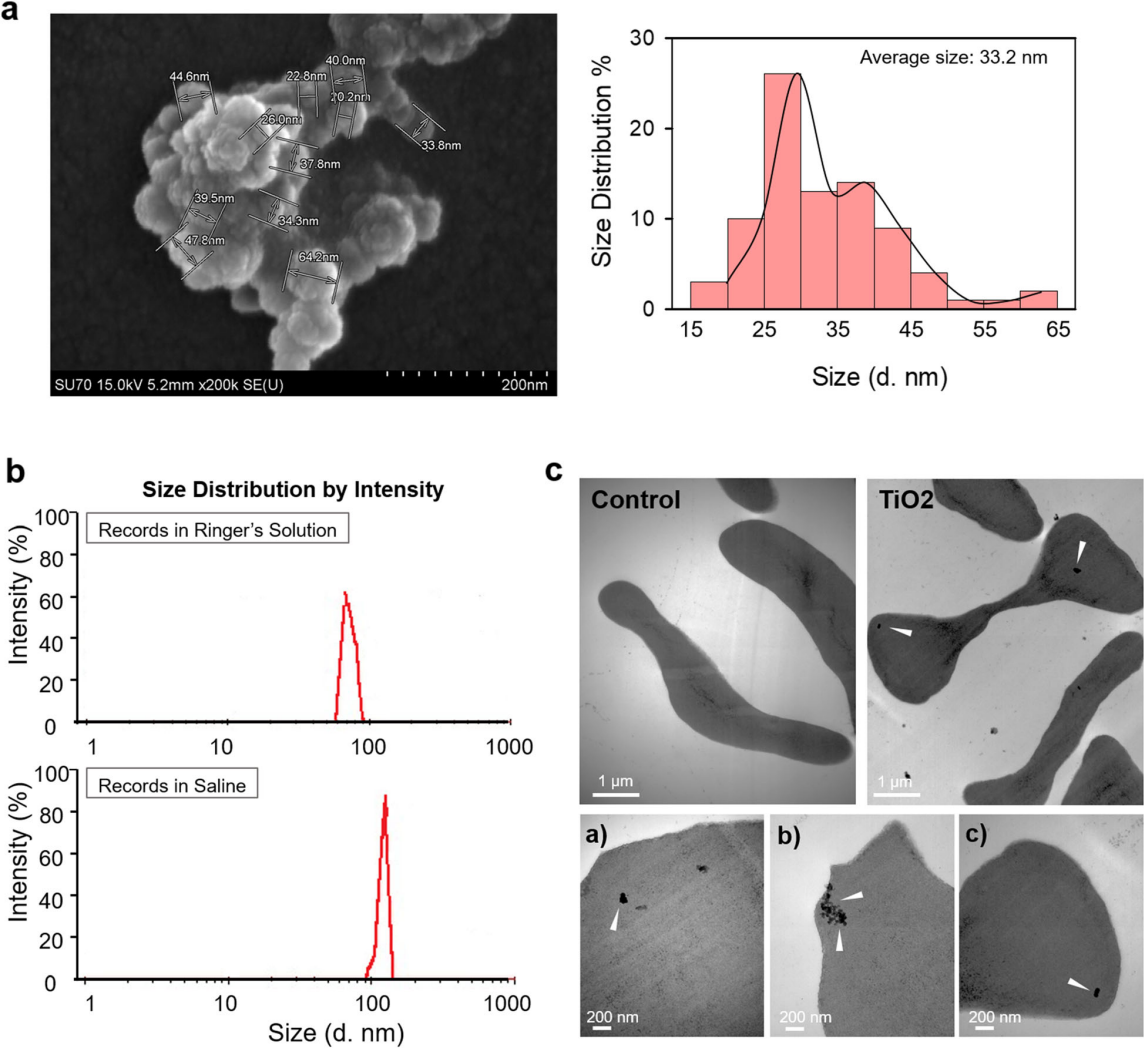
Characterization of TiO_2_ NPs and transmission electron microscope (TEM) observation of cellular uptake. (**a**) The size distribution histogram generated using SEM images showed TiO_2_ NPs of size between 15 and 65 nm with an average of 33.2 nm. Value are calculated from randomly measuring more than 100 particles of TiO_2_ NPs observed by SEM. (**b**) Particle size distribution of TiO_2_ NPs in Ringer’s solution used in vitro system and in saline used for intravenous injection showed particles of diameter with a peak of distribution at 68.1 nm and 122.4 nm, respectively (with 10% FBS in it). The results also indicated the presence of TiO_2_ NPs agglomerates both in Ringer’s solution and in saline. (**c**) Control (distilled water) and TiO_2_ NPs-treated RBCs were observed using TEM after 24 h treatment. The white arrowheads indicated TiO_2_ NPs might go through RBCs membranes and uptake by RBCs. Black scale bar: 2 μM. * represents significant differences from control group (*p* < 0.05)

The size distribution of TiO_2_ NPs was characterized with scanning electron microscopy (SEM) and dynamic light scattering (DLS). SEM observation showed that the majority of TiO_2_ NPs was at the size ranges of 20 to 45 nm with the average size of 33.2 nm as calculated with sampled one hundred particles (Fig. 1a). DLS data showed the peak and average size by intensity in Ringer’s solution (with 10% FBS) was 68.1 nm and 72.32 nm, respectively, and in saline (with 10% FBS), was 122.4 nm and 120.4 nm, respectively (Fig. 1b). In addition, the zeta potential of TiO_2_ NPs was − 8.70 mV in Ringer’s solution and - 10.58 mV in saline (pH 7.4). The physiochemical properties of TiO2 NPs were summarized in Supplemental Table S1. Next, we could also observe that TiO_2_ NPs penetrates through RBCs membranes and enter into RBCs using transmission electron microscopy (TEM) (Fig. 1c), which matched well the previous findings provided by Li, et al., and Rothen-Rutishauser, et al., [23, 24], indicating that TiO_2_ NPs exposure may produce significant biological or toxic effects on RBCs.

### Effects of TiO_2_ NPs on human isolated RBCs in vitro

**Fig. 2:**
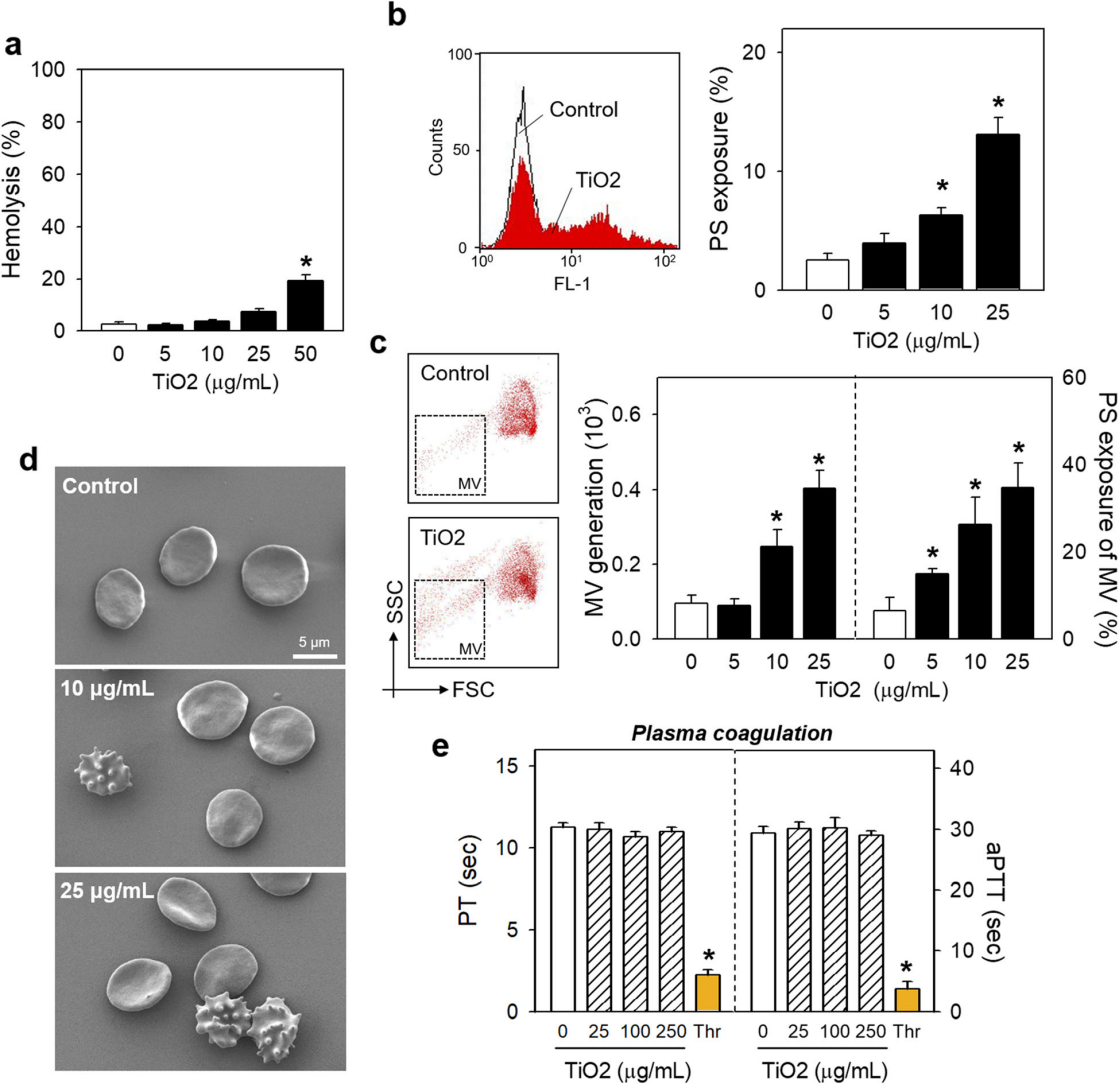
Effects of TiO2 NPs on human RBCs and plasma coagulation. (**a**) After isolated human RBCs were treated with various concentrations (0, 5, 10, 25 and 50 μg/mL) of TiO_2_ NPs at 37 °C for 24 h, the hemolytic activity was measured at 540 nm. (**b-d**) Isolated human RBCs were treated with various concentrations (0, 5, 10 and 25 μg/mL) of TiO_2_ NPs at 37 °C for 24 h. And then, (**b**) PS exposure and (**c**) MV generation were concentration-dependent promoted, which was determined using flow cytometry. (**d**) Morphological alteration was observed using SEM and echinocytes were concentration-dependently shown in TiO_2_ NPs-treated RBCs. (**e**) Plasma coagulation was evaluated by prothrombin time (PT) and activated partial thromboplastin time (aPTT) after human plasma was incubated with 0, 25, 100 and 250 μg/mL TiO_2_ NPs. Thrombin (1 U/mL) was used as positive control. Scale bar: 5 μm. Values are mean ± S.E. of 3–5 independent experiments, * represents significant differences from the control group (*p* < 0.05)

Firstly, we determined the hemolytic reactions of TiO_2_ NPs on human isolated RBCs and found that 50 μg/mL of TiO_2_ NPs caused a significant lysis while 0 ~ 25 μg/mL did not, where we continued our investigation in the following study (Fig. 2a). PS exposure and MV generation, key indicators of procoagulant activity of RBCs participating in thrombosis, were examined using flow cytometry [25]. Figure 2b showed that 10 to 25 μg/mL of TiO_2_ NPs treatment for 24 h significantly elicited PS exposure. The generation of PS-bearing MV (Fig. 2c) from TiO_2_ NPs-treated RBCs also increased in a concentration-dependent fashion. SEM observation showed the appearance of spiny cells, called echinocytes, in TiO_2_ NPs-treated groups (Fig. 2d). These findings reflect a well-known relationship between loss of phospholipid asymmetry and morphological changes [26]. Plasma coagulation, one of key events contributing to thrombosis, was estimated by measuring the prothrombin time (PT) and the activated partial thromboplastin time (aPTT), but no effects were induced by TiO_2_ NPs up to a level of 10 folds more than that exposed to RBCs (Fig. 2e), indicating the specificity in the effects of TiO_2_ NPs to RBCs. Indeed, compared to anatase TiO_2_ NPs, we futher determined hemolysis and PS exposure of rutile type and anatase/rutile mixture (Supplemental Figure 1), showing the toxicity was mixture > anatase > rutile.

### Effects of TiO_2_ NPs on phospholipid translocase, intracellular calcium level ([Ca^2+^]_i_), and caspase activity in RBCs

**Fig. 3:**
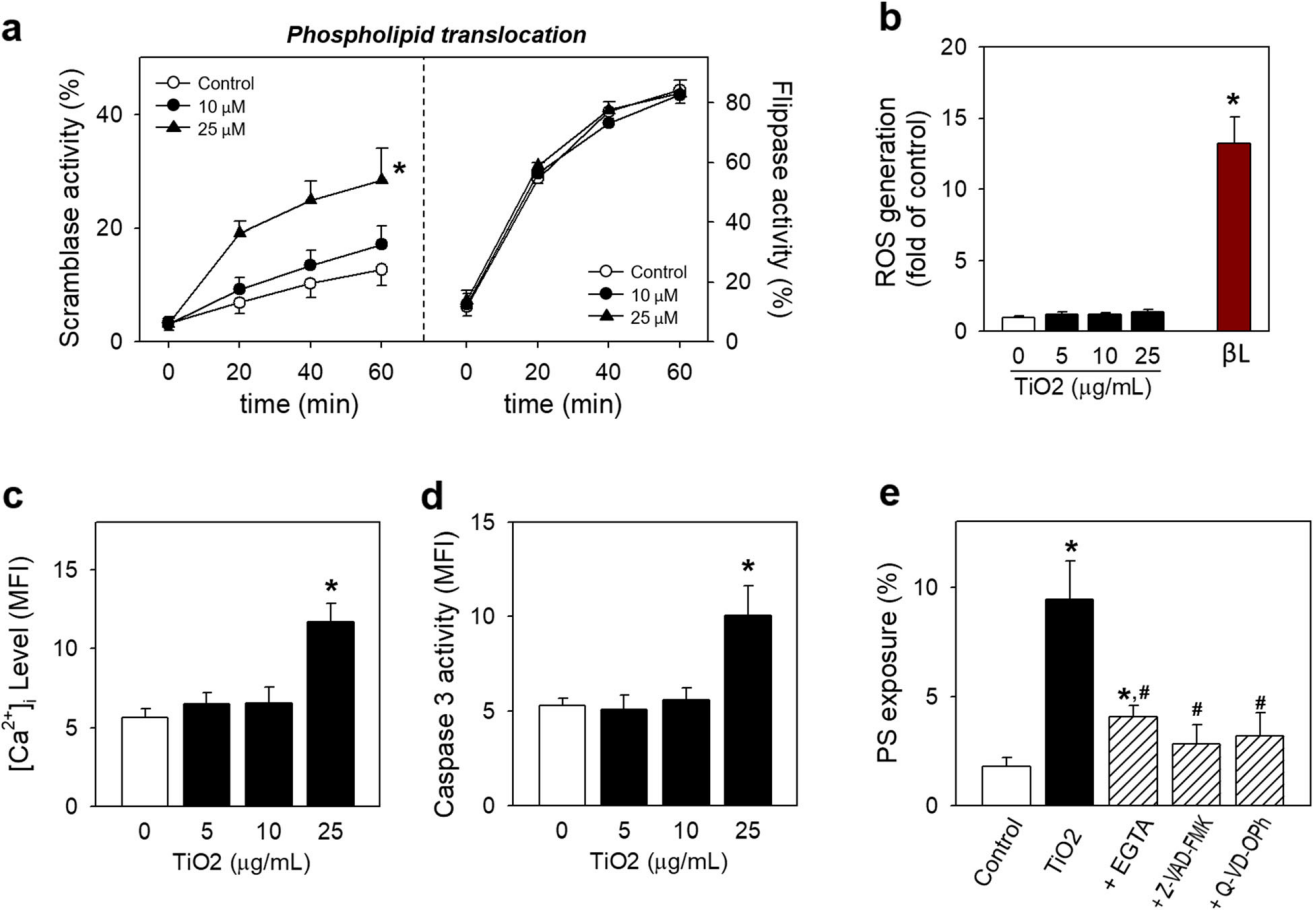
Intracellular events underlying PS exposure in RBCs induced by TiO_2_ NPs. Isolated human RBCs were treated with various concentrations (0, 5, 10 and 25 μg/mL) of TiO_2_ NPs at 37 °C for 24 h. Next, phospholipid translocation was shown by (**a**) concentration-dependently increased scramblase activity and (b) no changes of flippase activity. (**b**) ROS generation was determined through preloading 5 μM CM-H_2_DCF-DA for 30 min, and beta-lapachone was used as positive control. (**c**) Intracellular calcium, [Ca^2+^]_i_, was examined by preloading 3 μM fluo-4 AM for 1 h. (**d**) Caspase 3 activity was increased as measured shown in Method. (**e**) Inhibition of PS exposure was performed by preloading various inhibitors, a calcium chelating agent (5 mM EGTA) or caspase inhibitors (Z-VAD-FMK and Q-VD-Oph) for 3 h prior to exposure to TiO2 NPs for 24 h. Values are mean ± S.E. of 3–5 independent experiments, * represents significant differences from the control group (*p* < 0.05)

PS externalization is resulting from the disruption of phospholipid asymmetry, which is controlled by a balance of phospholipid translocases activity; scramblase and flippase [19]. After 24 h incubation with TiO_2_ NPs, scramblase activity was significantly upregulated in a concentration-dependent manner as evidenced by increased C6-NBD-PC translocation (Fig. 3a, left). On the contrary, TiO_2_ NPs exposure did not affect flippase activity as examined by absence of embedded C6-NBD-PS translocation (Fig. 3a, right), reflecting loss of phospholipid asymmetry leading to PS exposure induced by TiO_2_ was mainly attributable to the increased scramblase activity.

Scramblase activity was known to be increased by ROS [27, 28], intracellular calcium elevation [17] [29], and caspase-3 activation [30]. Compared to the positive control (Pb-treated RBCs) and negative control, no ROS generation was observed in RBCs following the treatment of TiO_2_ NPs (Fig. 3b). On the contrary, 25 μg/mL of TiO_2_ NPs resulted in increased [Ca^2+^]_i_ in RBCs (Fig. 3c). As well as increased [Ca^2+^]_i_, caspase-3 was significantly activated by TiO_2_ NPs exposure (Fig. 3d). The roles of increased [Ca^2+^]_i_ and activated caspase 3 in TiO_2_ NP-induced PS exposure in RBCs were further confirmed through pretreatment of their inhibitors, EGTA and Z-VAD-FMK, which succeeded to attenuate the PS exposure induced by TiO_2_ NPs treatment (Fig. 3e).

### Procoagulant activity of TiO_2_ NPs-exposed RBCs

**Fig. 4:**
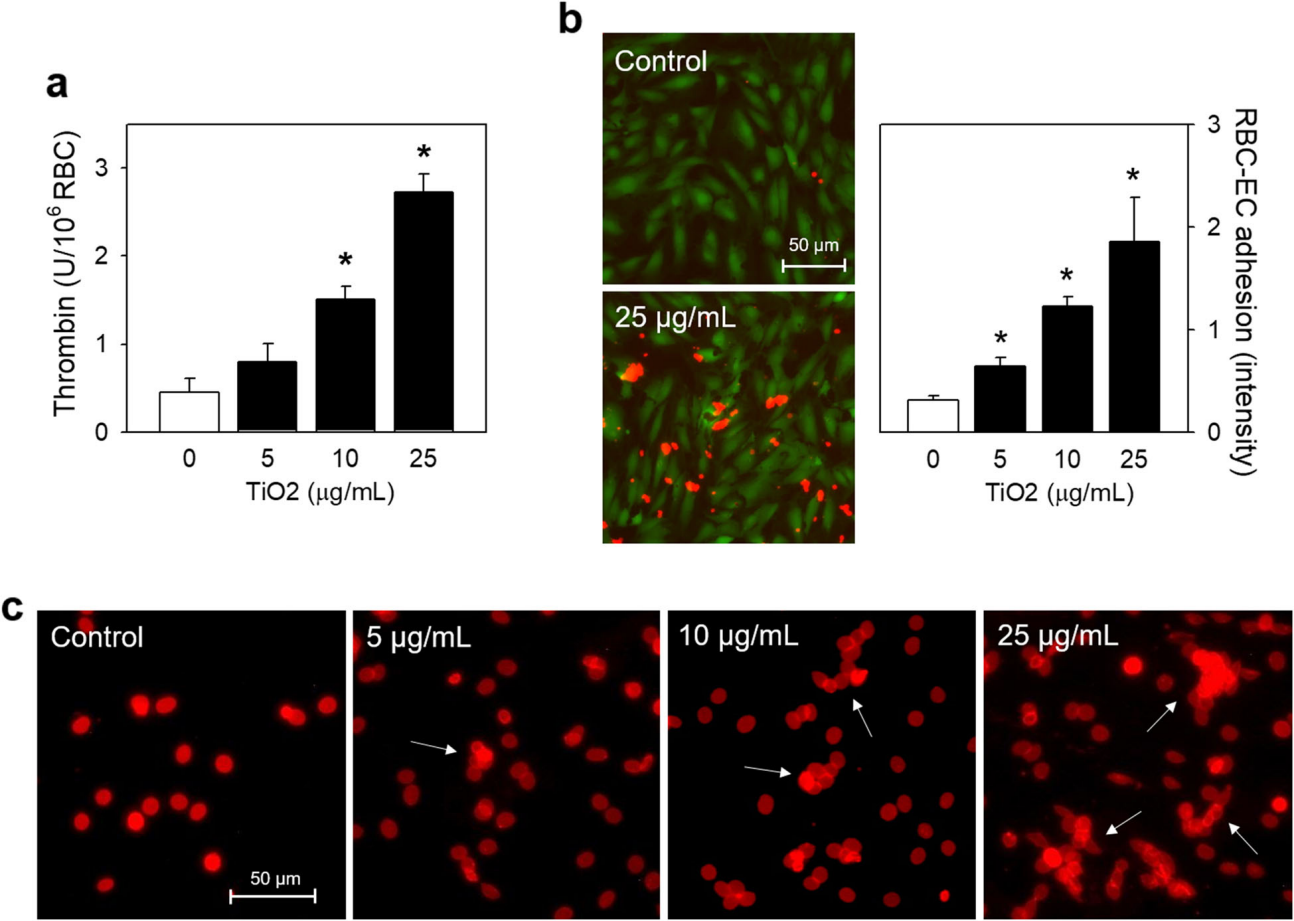
Biological functions of human RBCs after TiO_2_ NPs treatments. After isolated human RBCs were treated with various concentrations (0, 5, 10 and 25 μg/mL) of TiO_2_ NPs for 24 h at 37 °C, (**a**) procoagulant activity was shown by a concentration-dependent trend of thrombin generation using prothrombinase assay and (**b**) enhanced adherent RBCs to HUVECs was detected by fluorescence microscope as described in Methods. Endothelial cells (green fluorescence); RBCs (red fluorescence). Required further confirmation in the following study (white arrow). (**b,** right) The increased intensity shown as a bar graph was the fluorescence of red color, reflecting the relatively adherent RBCs to HUVECs. (**c**) RBC aggregation (white arrows) was initiated by 24 h treatment with TiO_2_ NPs as observed by fluorescence microscopy. Scale bar: 50 μm. Values are mean ± S.E. of 4 independent experiments, * represents significant differences from the control group (*p* < 0.05)

Procoagulant activity of RBCs results in accelerated thrombin generation, a key step for blood coagulation cascade, and the adhesion of RBCs to vascular wall, which could ultimately promote thrombosis [31]. Firstly, prothrombinase assay was performed to detect thrombin generated by TiO_2_ NPs-exposed RBCs. As a result, a concentration-dependent increase in thrombin generation was induced by TiO_2_ NPs as shown in Fig. 4a. It was well-matched with PS exposure and MV generation shown in Fig. 2b and c. Also, TiO_2_ NPs-exposed RBCs were more-adhesive to ECs with a concentration-dependent trend as observed by fluorescence microscopy shown in Fig. 4b. Of note, some red aggregates also appeared in TiO_2_ NPs-treated groups, suggestive of RBCs aggregation after TiO_2_ NPs exposure. Indeed, TiO_2_ NPs-exposed RBCs became more prone to aggregate in a concentration-dependent fashion (Fig. 4c).

### Prothrombotic effects of TiO_2_ NPs exposure in vivo

**Fig. 5:**
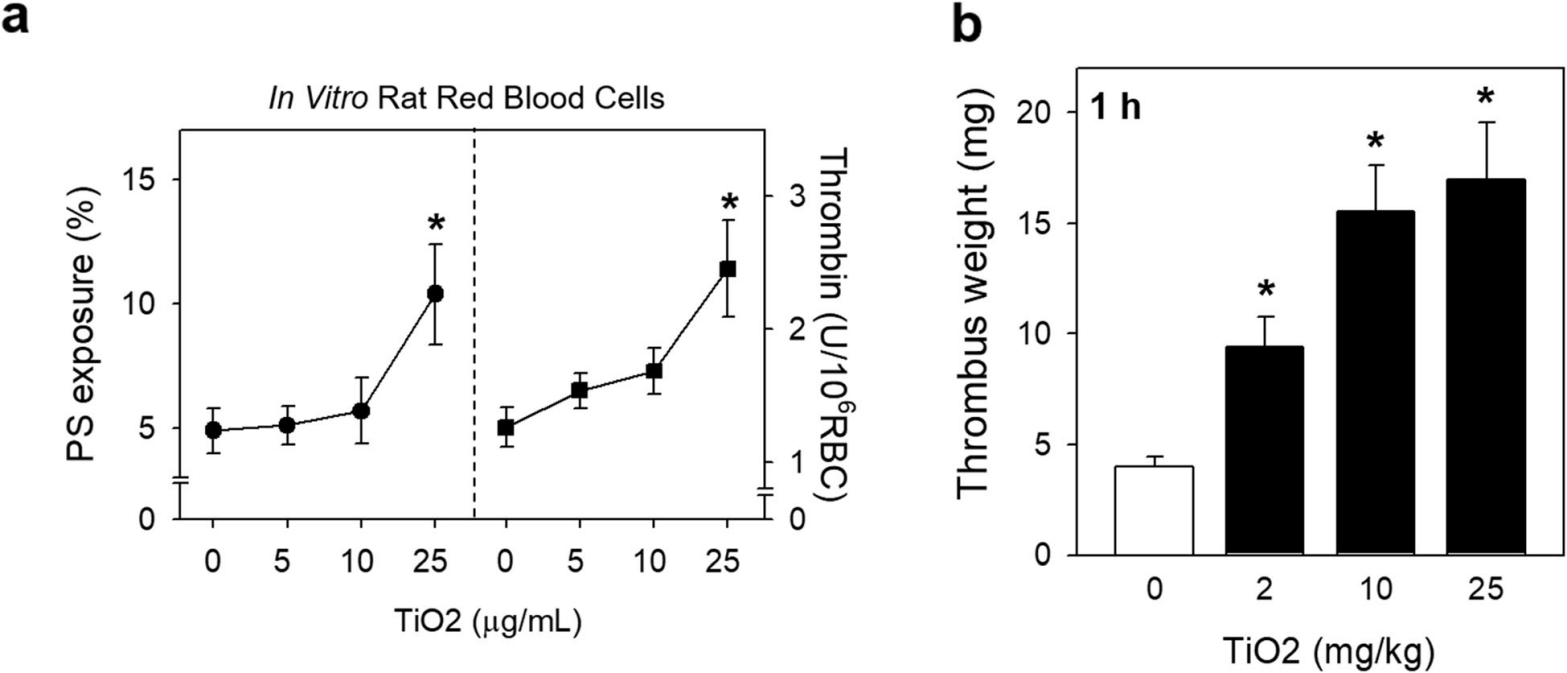
Thrombotic effects of TiO_2_ NPs exposure after intravenously injection to rat. (**a**) Blood was freshly collected from 8-week-old SD rats, and isolated rat RBCs were further incubated with various concentrations (0, 5, 10 and 25 μg/mL) of TiO_2_ NPs. (**a**, left) PS exposure as well as (**a**, right) thrombin generation were evaluated. (**b**) In a venous animal model, various doses of TiO_2_ NPs was intravenously injected to rats, and 1 h later, thrombus formation was assessed. Values are mean ± S.E. of 4–5 independent experiments, * represents significant differences from the control group (*p* < 0.05)

**Scheme 1:**
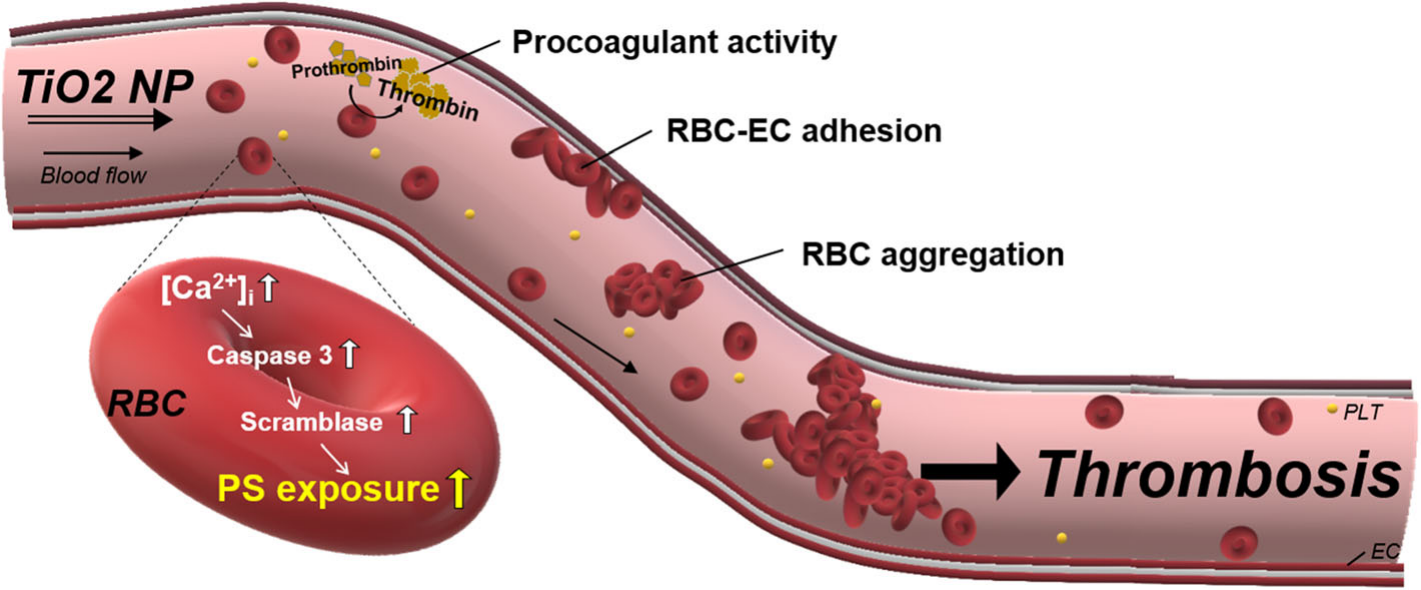
Venous Thrombosis induced by TiO_2_ NPs via RBCs.

Prior to in vivo assessment of TiO_2_ NPs in rats, a bridge study was performed using freshly isolated rats RBCs to confirm the procoagulant effects of TiO_2_ NPs on rat RBCs. Consistently with human RBCs, rat RBCs exposed to TiO_2_ NPs showed a concentration-dependent increase in PS exposure and thrombin generation (Fig. 5a) (Scheme 1). Next we examined whether TiO_2_ NPs exposure could elicit thrombosis using venous thrombosis rat model 1 h after TiO_2_ NPs were intravenously injected (0, 2, 10, 25 mg/kg) to rats. On average, rats have around 64 ml of blood per kg of bodyweight [32]. And when we injected 2 mg/kg of TiO_2_ NPs, there will be around 30 μg/mL in rat blood, which matched well with in vitro concentrations exposed to isolated blood. As a result, thrombus formation was significantly increased by TiO_2_ NPs in a dose-related fashion (Fig. 5b), confirming the thrombotic risks of TiO_2_ NPs.

## Discussion

In this study, we demonstrated that titanium dioxide nanoparticles (TiO_2_ NPs) could initiate phosphatidylserine (PS) exposure and promote procoagulant activity in isolated human red blood cells (RBCs) and rat RBCs. Here, intracellular calcium increase and caspase 3 activity up-regulated scramblase activity leading to loss of phospholipid asymmetry and PS exposure in TiO_2_ NPs-treated RBCs. Furthermore, TiO_2_ NPs led to accelerated thrombin generation, RBC-EC adhesion as well as RBC aggregation, and more importantly, could increase thrombus formation in rats in vivo supporting the relevance of our findings to real in vivo states.

Previous studies showed that TiO_2_ NPs can induce toxicity in various cells and tissues in vitro, such as lymphocytes, platelets and liver tissues, but their pathophysiological implications remained unexplored [6, 7, 10, 33, 34]. In RBCs, hemolysis is repeatedly observed as TiO_2_ NP-induced toxicity in several studies [7, 24, 35], but its pathophysiological significance is poorly understood. Recently, Li et al. have observed that TiO_2_ NPs adhere to RBC membranes and induce the morphological alterations [24]. In this study, we demonstrated that the TiO_2_ NPs can induce procoagulant activation of RBCs both in vitro and in vivo systems. We also elucidated its underlying mechanisms and explored its further biological significance in terms of thrombosis, providing with a comprehensive and convincing evidence on the prothrombotic effects of TiO_2_ via the procoagulant activity of RBCs.

TiO_2_ NPs are applied via intravenous administration for their medical uses [36], raising the necessity of the careful and rigorous safety assessment of TiO_2_ application in vivo. An earlier study investigated TiO_2_ toxicity in mice using an extremely high dosage (0, 140, 300, 645, or 1387 mg/kg), found diverse degrees of dysfunction in the brain, lung, spleen, liver and kidneys [37]. Another study demonstrated that i.v. injection of 5 mg/kg of TiO_2_ to rats were without detectable toxicity, and suggested a safe level of TiO_2_ up to 5 mg/kg [38]. But these studies appear to focus the general toxicology, leaving subtle pathophysiological effects unaddressed. Our study showed that TiO_2_-injection at 2 mg/kg i.v can provoke thrombosis, suggesting that careful attention shall be paid for pathophysiological effects of TiO_2_ NPs as well to ensure the safety.

In addition, bio-distribution after i.v. injections of TiO_2_ NPs in rats investigated in several studies revealed that a majority of TiO_2_ NPs distributes in blood followed by spleen, liver and lung [38–40]. A recent study showed that after i.v. administration of 0.95 mg/kg TiO_2_ NPs in rats, the blood level of 420 ng/mL was observed at 5 min, which is similar to the range employed in our studies in vitro and in vivo. Moreover, the half-life of TiO_2_ NPs was determined to be very long ranging up to 12.56 days in rat after giving i.v injection [39], suggesting that stronger prothrombotic effects of TiO_2_ NPs could be anticipated, although further studies are necessary to confirm it.

Oxidative stress accompanied by decreased glutathione and overproduced ROS has been frequently involved in the toxicity of TiO_2_ NPs exposure [41, 42]. In contrast, we found no obvious production of ROS in TiO_2_ NPs-treated RBCs at any concentrations, which is in line with a previous finding that intracellular glutathione levels in blood, lung and liver cells were not affected by TiO_2_ NPs [43]. Instead, we newly found that intracellular calcium and caspase-3 activation are elicited after TiO_2_ NPs exposure, revealing new molecular targets for the toxicity of TiO_2_ NPs. We suggest that studies are necessary to identify the role of intracellular calcium increase and caspase-3 activation in other pathological effects of TiO_2_ NPs in the near future.

Various TiO2 NPs possess distinct physicochemical properties, such as particle sizes, crystalline forms (anatase or rutile phase), surface modification (surface charge and coating), and protein corona formation, each of which would be expected to substantially affect their biological properties. It is well established that the anatase form of TiO2 is more bioactive than rutile type which, together with smaller size, can result in greater toxicity [6, 44]. Also, the surface functionalization of NPs with negatively charged groups could alleviate the erythrocyte aggregating effects of these NPs [45], which could be attributable to the formation of a complex system on the NP surface induced by surface modification. Exemplifying this, a previous study showed that BSA-coated gold NPs induce significantly lower hemolysis [46]. NP-protein corona complex formation could also affect the biological properties of NPs [47]. A previous study demonstrated that the formation of plasma protein corona on NP surface protects RBCs from both hydrophilic and hydrophobic NP-mediated hemolysis [48]. Against this backdrop, we believe that it would be interesting to examine the effects of various TiO2 NPs for thrombotic risk mediated by RBCs in the future**.**

## Conclusion

Our study revealed the prothrombotic effect of TiO_2_ NPs via the procoagulant activity of RBCs, which we demonstrated with in vitro and in vivo assessments. We could also propose the mechanism underlying by showing that TiO_2_ NPs-induced PS exposure in isolated human RBCs was related to increased [Ca^2+^]_i_ level and caspase 3 activity but independent of ROS generation. Most importantly, we demonstrated the in vivo relevance of our findings through showing that TiO_2_ NPs exposure can increase thrombosis in rat venous thrombosis model in vivo, reflecting the need of an attention during medical use of TiO_2_ NPs.

## Methods

### Materials

The following chemicals were purchased from Sigma Chemical Co. (St. Louis, MO): TiO_2_ (titanium dioxide, anatase, nanopowder, < 25 nm particle size, 99.7% trace metals basis.), L-ascorbic acid, N-acetylcysteine, glutaldehyde solution and osmium tetroxide, CaCl_2_, glucose, ethylenediaminetetraacetic acid (EDTA), bovine serum albumin (BSA), N-[2Hydroxyethyl]piperazine- N´-[2-ethanesulfonic acid] (HEPES), sodium dodecyl sulfate and purified human thrombin. Phycoerythrin-labeled monoclonal mouse anti-human CD235a and fluorescein-isothiocyanate (FITC)-labeled annexin V (annexin V-FITC) were from BD Pharmingen (San Diego, CA). Fluo-4 acetoxymethyl ester (fluo-4 AM) and the chloromethyl derivative of 2′, 7′-dichlorodihydrofluorescein diacetate (CM-H_2_DCF-DA) were obtained from Life Technologies. 1-Palmitoyl-2-[6-[(7-nitro-2-1,3-benzoxadiazol-4-yl)amino]hexanoyl]-snglycero-3-phospho-L-serine (C_6_-NBD-PS) and 1-oleoyl-2-[6-[(7-nitro-2-1,3-benzoxadiazol-4-yl)amino]hexanoyl]-sn-glycero-3-phosphocholine (C_6_-NBD-PC) were from Avanti Polar Lipids (Alabaster, AL). Caspase 3 detection kit (FITC-DEVE-FMK) was purchased from Calbiochem (San Diego, CA). Caspase-3 inhibitor II and Q-VD-OPh were obtained from Merck Millipore. Purified human prothrombin, factor Xa and factor Va were from Hematologic Technologies, Inc. (Essex Junction, VT), and S2238 was from Chromogenix (Milano, Italy). Human umbilical vein endothelial cells (HUVECs) and the endothelial cell growth media (EGM) kit were purchased from Lonza. Calcein-green AM was from Invitrogen (Carlsbad, CA). Human recombinant tissue factor (Recombiplastin) was obtained from Instrumentation Laboratory (Lexington, MA) and thromboplastin (Simplastin Excel) was from Biomerieux (Durham, NC).

### Preparation of RBCs

With the approval from the Ethics Committee of Health Service Center at Seoul National University, we collected blood from healthy volunteers. As gender, age and diseases are all risk factors of thrombosis, we only used healthy male donors ranging from 20 to 30 years old to simplify our study design. Human blood was collected using a vacutainer with acid citrate dextrose (ACD) and a 21 gauge needle (Becton Dickinson, U.S.A.) on the day of each experiments. Platelet rich plasma and buffy coat were removed after centrifugation at 200 g for 15 min. Packed RBCs were washed twice with phosphate buffered saline (PBS: 1.06 mM KH_2_PO_4_, 154 mM NaCl and 2.96 mM Na_2_HPO_4_ at pH 7.4) and once with Ringer’s solution (125 mM NaCl, 5 mM KCl, 1 mM MgSO_4_, 32 mM HEPES, 5 mM glucose, pH 7.4). Washed RBCs were resuspended in Ringer’s solution to a final cell concentration of 5 × 10^7^ cells/mL with 1 mM CaCl_2_ before use.

### Characterization of TiO_2_ NPs

TiO_2_ NPs were purchased from Sigma Aldrich (reference 637,254). They are nano powder, < 25 nm particle size and 99.7% trace metals basis. All heavy metal impurities detected in the sample were equal to/under 65 ppm via XRF. The density is 3.9 g/ml (relative density) and 0.04–0.06 g/mL (bulk density). Fusion temperature was determined as 1825 °C and specific surface area as 44–55 m^2^/g via BET. It is not surface treated. Sigma did not test the solubility or dissolution of this product. However, these products are insoluble in water, HCl, HNO_3_ and dilute H_2_SO_4_. It is soluble in hot concentrated H_2_SO_4_ and hydrofluoric acid (supplier data). Preparation of TiO_2_ NPs suspension was carried out according to the methods previously described. TiO_2_ NPs were dispersed in distilled water as 100 X stock solution (1–25 mg/mL) and sonicated with a probe type sonicator with a maximum output power, 200 W (Branson Sonifier, Danbury, CT) for 30 s to prevent particles self-assembly (agglomeration) prior to each experiment. For the characterization of TiO_2_ NPs, TiO_2_ NPs was dried and was observed with scanning electron microscope (SEM) (ZEISS, MERLIN Compact) to examine the size distribution. A detailed statistical analysis of TiO_2_ NPs was performed by randomly measuring 100 nanoparticles and the procedure was operated by manually outlining the particles from several images taken by SEM. The hydrodynamic diameter and the zeta potential of the nanoparticles were measured by dynamic light scattering (DLS-7000, Otsuka Electronics, Co., Osaka, Japan) and electrophoretic light scattering (ELSZ-1000 Photal; Otsuka Electronics,Co., Osaka, Japan), respectively.

Cellular uptake of TiO_2_ NPs by RBCs was observed using TEM after the following procedures. After 24 h incubation of isolated RBCs with distilled water (as control) and 25 μg/mL of TiO_2_ NPs dispersed as a colloidal suspension in distilled water, 2% glutaraldehyde solution was used for cell fixation in the refrigerator for 1 h and 1% osmium tetroxide was used for post-fixation for 2 h. After en-bloc staining with 0.5% uranyl acetate for 30 min, serially dehydration was done with 30, 50, 70, 80, 90% (1 time) and 100% ethanol (3 times). Next, transition and infiltration was gradually done with propylene oxide (10 min, 2 times), once with propylene oxide and spurr’s resin (1,1) for 2 h, and spurr’s resin in the desiccator overnight. On the next day, infiltration was completed with newly spurr’s resin for 2 h in the desiccator, then samples were kept in the 70 °C oven overnight for polymerization. Finally, samples were observed under TEM (JEOL, JEM 1010).

### Evaluation of hemolysis

After incubation with TiO_2_ NPs, samples were centrifuged (10,000 g for 1 min) and the extent of hemolysis was determined spectrophotometrically at 540 nm. Ringer’s solution and RBCs lysed with triton X-100 were used as blank and 100% hemolysis, respectively.

### Flow cytometric analysis

Annexin V-FITC and anti-glycophorin A-PE were used for PS detection and RBC identification, respectively. Negative controls for annexin V binding were in the presence of 2.5 mM EDTA instead of 2.5 mM CaCl_2_. Flow cytometer FACS Calibur (Becton Dickinson, U.S.A.) equipped with an argon-ion laser emitting at 488 nm was applied for sample analysis. Data from 5000 events were collected and analyzed using Cell Quest Pro software. PS were identified by forward scatter characteristics after calibration by 1% standard beads. Both PS exposure in RBC area and MV area could be analyzed.

For determination of phospholipid translocation, 0.5 μM C_6_-NBD-PC (for scramblase activity) and C_6_-NBD-PS (for flippase activity) were added to TiO_2_ NPs-activated RBCs, respectively, for various durations (0, 20, 40 and 60 min) at 37 °C. The amount of internalized probe was determined by comparing the fluorescence intensity associated with the cells before (without 1% bovine serum albumin) and after (with 1% bovine serum albumin) back-extraction on ice for 10 min.

With flow cytometry, ROS generation and intracellular calcium ([Ca^2+^]_i_) were performed by determination of the fluorescence of intracellular DCF with pre-loading 5 μM CM-H_2_DCF-DA with RBCs (30 min, 60 rpm, 37 °C water bath, dark) and the fluorescence of fluo-4 with pre-loading 3 μM Fluo-4 AM with RBCs (1 h, 60 rpm, 37 °C water bath, dark) before TiO_2_ NPs treatments.

As well, caspase-3 activity was measured by post-adding 1 μL FITC-DEVD-FMK (a caspase-3 inhibitor conjugated to FITC as the fluorescent in situ marker) to 300 μL TiO_2_ NPs exposed-RBCs suspension (37 °C, 1000 rpm, dark). Re-suspended cells after centrifugation (1000 g for 5 min) and twice washing was detected using flow cytometry. Data from 5000 events were collected and analyzed using Cell Quest Pro software (Becton Dickinson).

### Morphological alteration observation using scanning electron microscopy (SEM)

After incubation with TiO_2_ NPs, RBCs were pre-fixed with 2% glutaraldehyde solution for 1 h at 4 °C and post-fixed with 1% osmium tetroxide for 30 min at room temperature in the hood. Then, samples were dehydrated serially with 50, 70, 80, 90, and 100% ethanol. After drying and coating with gold, the morphological alteration were observed on a SEM.

### Experiments with plasma

Platelet-poor plasma (PPP) was obtained from the precipitated fraction of PRP by centrifugation for 20 min at 2000 g. In PPP, PT and aPTT were measured in BBL Fibrometer (Becton Dickinson, Cockeysville, Maryland), based upon the procedures in PT and aPTT reagent kit, respectively.

### Prothrombinase assay

After incubation with TiO_2_ NPs for 24 h, samples were incubated with 5 nM factor Xa and 10 nM factor Va in Tyrode buffer (134 mM NaCl, 10 mM HEPES, 5 mM glucose, 2.9 mM KCl, 1 mM MgCl_2_, 12 mM NaHCO_3_, 0.34 mM Na_2_HPO_4_, 0.3% BSA, and 2 mM CaCl_2_ at pH 7.4) for 3 min at 37 °C. Thrombin formation was initiated by adding 2 μM prothrombin. Exactly 3 min after adding prothrombin, an aliquot of the suspension was transferred to a tube containing stop buffer (50 mM Tris-HCl, 120 mM NaCl, and 2 mM EDTA at pH 7.9). Thrombin activity was determined using the chromogenic substrate S2238 (chromogenic substrate for thrombin; Chromogenix, Milano, Italy). We calculated the rate of thrombin generation from the change in absorbance at 405 nm using a calibration curve generated with active-site–titrated thrombin.

### Observation under fluorescence microscope

Endothelial cells (2 × 10^4^ cells) were seeded in a 4-well-chamber for 2 days and stained with calcein green for 20 min. TiO_2_ NPs-treated RBCs were washed once and resuspended in EBM-2 to a final cell concentration of 5 × 10^7^ cells/mL. After HUVECs were washed twice with EBM-2, TiO_2_ NPs-exposed RBCs were layered onto confluent HUVEC monolayer and incubated for 60 min at 37 °C. After the incubation, the chambers were rinsed once with EBM-2 to remove non-adherent RBCs, and glycophorin A-PE were added for staining RBCs. Adhered RBCs to HUVECs were observed using fluorescent microscopy.

Moreover, aggregation of chemical-exposed RBCs was observed using fluorescence microscopy after adding glycophorin A-PE.

### In vivo assessment

Sprgue-Dawley (SD) rats (male, 300–400 g) were anesthetized with urethane (1.25 g/kg, i.p.). Blood (3.8% sodium citrate) was collected from abdominal aorta and RBCs were isolated as human RBCs preparation. Isolated rat RBCs were further incubated with TiO_2_ NPs for 24 h, then PS exposure and procoagulant activity were determined mentioned above.

In a thrombosis animal model, we surgically opened the abdomen and carefully dissected to expose the vena cava. A 16 mm apart around the vena cava was prepared with two pieces of loose cotton threads each side and we ligated all side branches tightly with cotton threads. Here, the NPs were suspended in saline (0.9% NaCl) for intravenous injection. 1 h after intravenously injecting TiO_2_ NPs (0, 2, 10 or 25 mg/kg) into a left femoral vein, we infused 500-fold diluted thromboplastin for 1 min to induce thrombus formation. Stasis was initiated by tightening the two threads, first the proximal and the distal thereafter. The abdominal cavity was provisionally closed, and blood stasis was maintained for 15 min. After reopening the abdomen, the ligated venous segment was excised and opened longitudinally to remove the thrombus. The isolated thrombus was blotted of excess blood and immediately weighed.

### Statistical analysis

The means and standard errors of means were calculated for all treatment groups. The data were subjected to two-way analysis of variance followed by Duncan’s multiple range test or student t test to determine which means were significantly different from the control. In all cases, a *p* value of < .05 was used to determine significant differences.

## Supplementary Information

**Additional file 1 Table S1.** Summary of physicochemical properties of TiO_2_ NPs.

**Additional file 2 Figure S1. Comparison of anatase, rutile and anatase/rutile mixture TiO**_**2**_ **NPs on hemoytic response and PS exposure in human isolated RBCs.** (a) Hemolysis and (b) PS exposure of human isolated red blood cells was determined after 24 h exposure to 50 μg/mL of each types of TiO2 NPs including anatase, rutile (Sigma 637,262, nanopowder, < 100 nm particle size via BET, 99.5% trace metals basis) and anatase/rutile mixture (Sigma 634,662, < 100 nm particle size via BET, 99.5% trace metals basis). Values are mean ± S.E. of 3–5 independent experiments, * represents significant differences from the control group (*p* < 0.05).

## Data Availability

Yes
