# Supplementary figures and images for: Titanium dioxide nanoparticles enhance thrombosis through triggering the phosphatidylserine exposure and procoagulant activation of red blood cells
Source: Part Fibre Toxicol. 2021 Aug 4;18:28. doi: 10.1186/s12989-021-00422-1 (PMC8336274; doi:10.1186/s12989-021-00422-1)

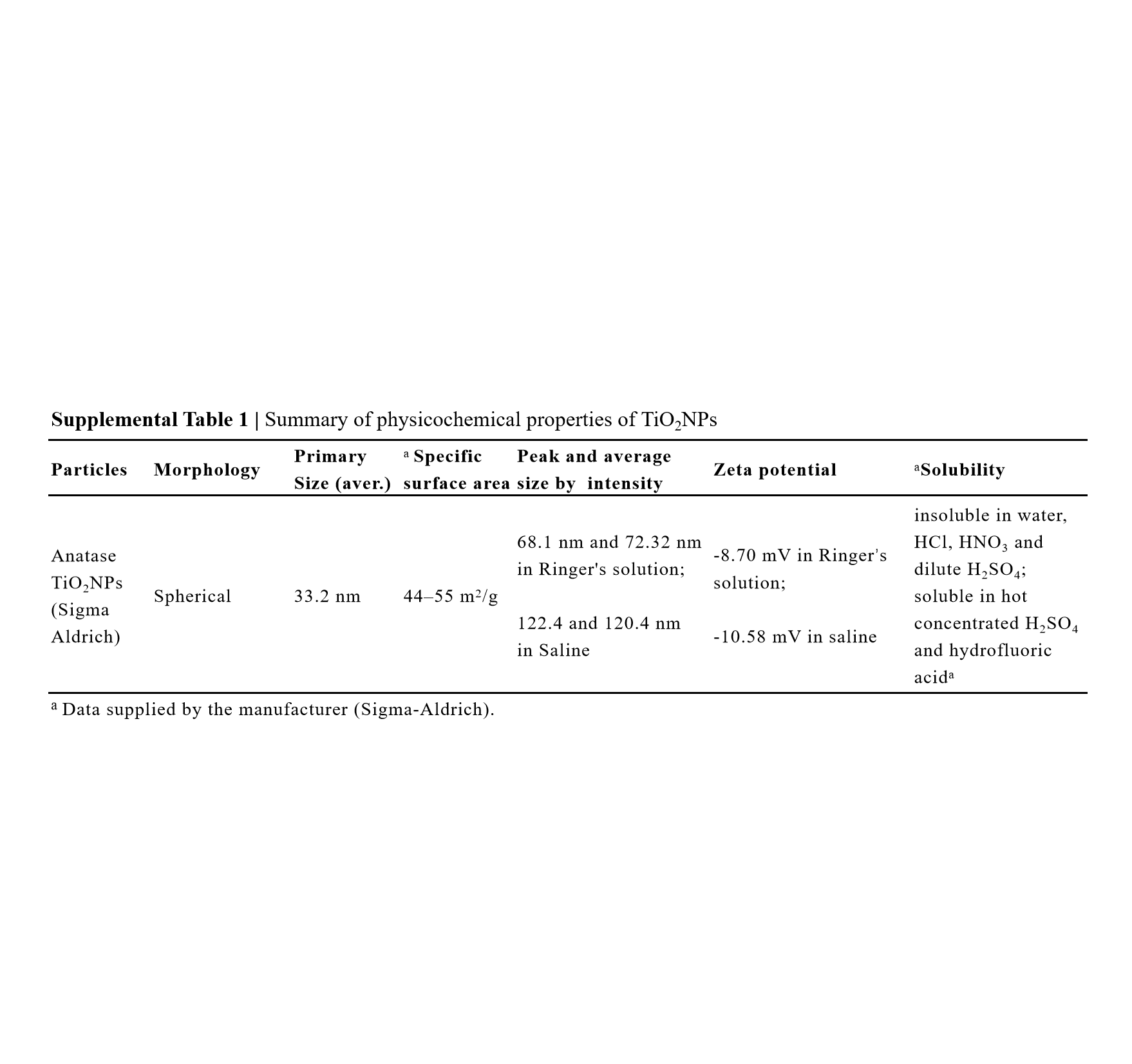

Supplement: Supplementary file 1 — Additional file 1 Table S1. Summary of physicochemical properties of TiO2 NPs. [file 12989_2021_422_MOESM1_ESM.tif]

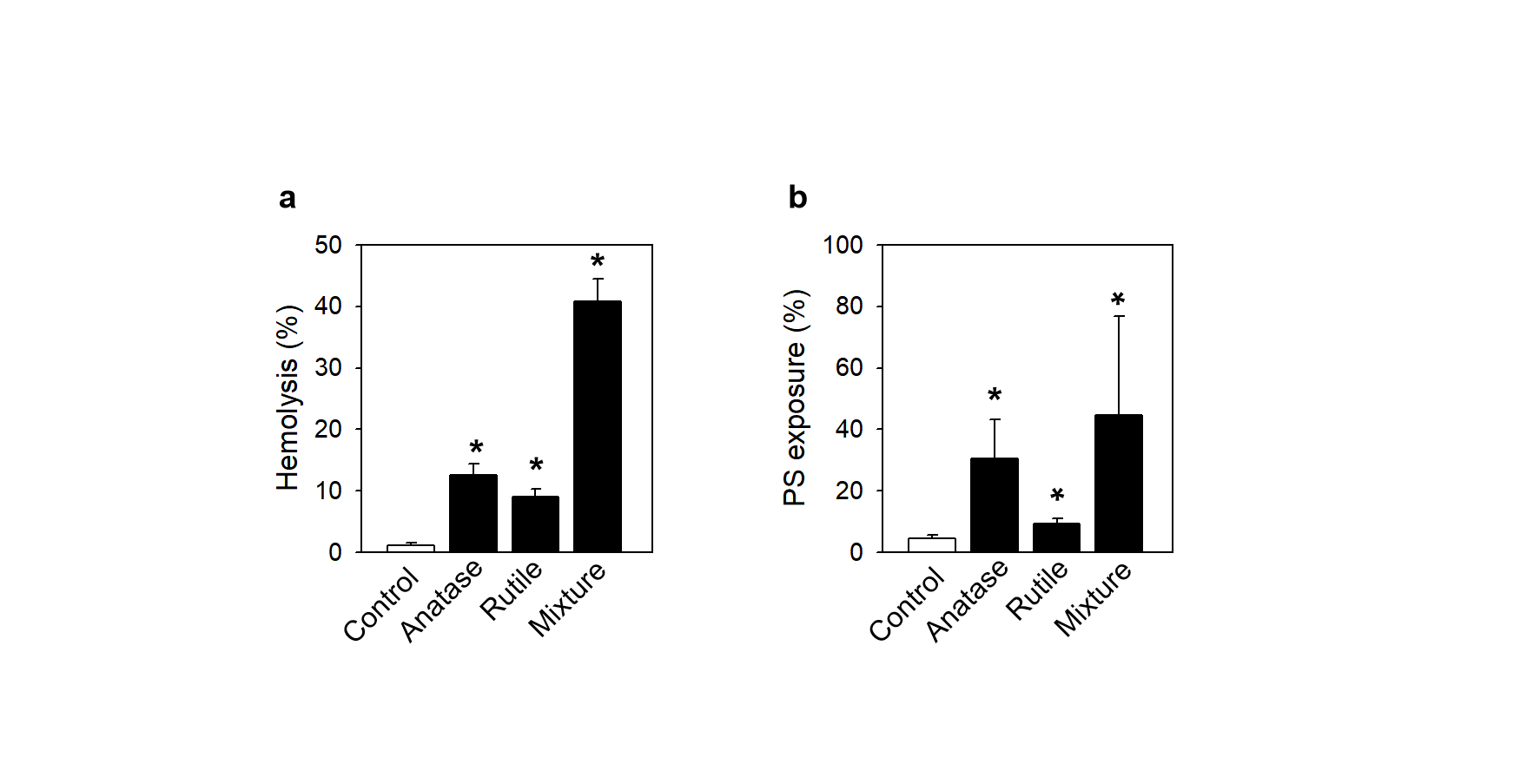

Supplement: Supplementary file 2 — Additional file 2 Figure S1. Comparison of anatase, rutile and anatase/rutile mixture TiO2 NPs on hemoytic response and PS exposure in human isolated RBCs. (a) Hemolysis and (b) PS exposure of human isolated red blood cells was determined after 24 h exposure to 50 μg/mL of each types of TiO2 NPs including anatase, rutile (Sigma 637,262, nanopowder, < 100 nm particle size via BET, 99.5% trace metals basis) and anatase/rutile mixture (Sigma 634,662, < 100 nm particle size via BET, 99.5% trace metals basis). Values are mean ± S.E. of 3–5 independent experiments, * represents significant differences from the control group (p < 0.05). [file 12989_2021_422_MOESM2_ESM.tif]
